# Impact of extrathyroidal autoimmune diseases on clinical features and the efficacy of Iodine‐131 therapy in patients with differentiated thyroid cancer

**DOI:** 10.1002/iid3.70018

**Published:** 2024-09-25

**Authors:** Ya‐hong Long, Na Li, Le Ma, Wan‐chun Zhang

**Affiliations:** ^1^ Department of Nuclear Medicine, Shanxi Bethune Hospital, Shanxi Academy of Medical Sciences Third Hospital of Shanxi Medical University, Tongji Shanxi Hospital Taiyuan China

**Keywords:** ^131^I, autoimmune disease, clinical features, differentiated thyroid cancer, efficacy

## Abstract

**Objective:**

The aim of this study is to assess the impact of extrathyroidal autoimmune diseases (ADs) on the clinical characteristics and efficacy of iodine‐131 (^131^I) therapy in patients with differentiated thyroid cancer (DTC).

**Methods:**

Patients with DTC who were received ^131^I therapy simultaneously were classified into the combination group (*n* = 35) and noncombination group (*n* = 146) depending on the presence of ADs. The clinical characteristics, such as gender, age, tumor lesions, lymph node metastasis, distant metastasis, ^131^I therapy efficacy, and use of levothyroxine, were compared between the two groups. Statistical analysis was conducted using SPSS 26.0 and R 4.0.3.

**Results:**

There was a statistically significant difference in age between the combination and noncombination groups (*t* = −2.872, *p* < .01), and the optimal cutoff value was 50.5 years. Propensity score matching was completed effectively on a total of 121 patients, using age as the matching factor, comprising 32 cases in the combination group and 80 cases in the noncombination group. The baseline demographic features of the two groups were equivalent after matching (*p* > .05), and there was no significant difference in the therapeutic efficacy of ^131^I between the two groups (*p* > .05). In the subgroup analysis involving patients aged great than 50.5 years, the levothyroxine/weight (µg/kg) was increased in the combination group, and the difference was statistically significant (*p* < .05).

**Conclusion:**

While extrathyroidal ADs may enhance the detection of DTC among elderly women, they have no impact on the clinical characteristics of thyroid cancer or the efficacy of ^131^I therapy. ADs may necessitate higher per‐unit dosages of levothyroxine in patients with DTC, regardless of the clinical status. Consequently, it is not essential for nuclear medicine physicians to consider the presence of ADs when designing treatment plans for patients with DTC.

## 1 INTRODUCTION

Thyroid cancer is the most prevalent endocrine tumor and its incidence is rising annually worldwide.^1^, ^2^ There is consensus that the increased incidence of thyroid cancer is associated with the increased detection of more small lesions. Studies of the molecular pathogenesis of well‐differentiated and poorly differentiated thyroid cancer (DTC) have revealed that inflammation and the immune system play a role in thyroid tumorigenesis.^3^ Based on a vast number of clinical studies, Hashimoto's thyroiditis (HT) and papillary thyroid carcinoma (PTC) are strongly associated, and the prevalence of DTC combined with HT is increasing.

Numerous similarities exist between the two conditions, such as readily observable perifocal lymphocyte infiltration or lymphocytic thyroiditis lesions within thyroid tissues. Also, shared features encompass soluble mediators (including chemokines, cytokines, and growth factors) and the presence of positive rearranged during transfection (RET)/PTC rearrangement. These commonalities offer promising avenues for assessing the relationship between the two disorders and developing more effective treatment methods for thyroid cancer. As reported, patients with DTC complicated by HT represent a notable proportion, ranging from 12.0% to 43.8% of all DTC cases.^4^, ^5^ HT poses a paradoxical dilemma for patients with thyroid cancer, as it increases the susceptibility to thyroid cancer, particularly PTC, while concurrently impeding the progression of PTC. Patients exhibiting tumor‐associated lymphocytes present with advanced disease stages and an increased incidence of invasion and lymph node metastasis when compared to counterparts lacking tumor‐associated lymphocytes or HT.^6^, ^7^

Autoimmune diseases (ADs) and malignant tumors represent two significant threats to human health and longevity. There is a close association between ADs and malignancies, with patients with ADs exhibiting a higher propensity for cancer development when compared to the general population. Notably, patients with autoimmune conditions such as rheumatoid arthritis (RA), systemic lupus erythematosus (SLE), and Sjogren's syndrome (SS) demonstrate an increased risk of malignant tumors.^8^ Conversely, malignant tumors can provoke manifestations resembling ADs, such as RA‐like syndrome, inflammatory myositis, and vasculitis.^9^ ADs have been implicated in influencing the onset of thyroid cancer, with studies indicating an increased risk of thyroid malignancy in patients with RA,^10^ SLE,^11^ SS,^12^ inflammatory bowel disease,^13^ and vitiligo.^14^ A recent prospective multicenter study further substantiated these findings, revealing that individuals with either organ‐specific or systemic ADs have a higher risk of thyroid cancer when compared to the general population.^15^ Despite the recognized association, there is a lack of research examining the manifestations, clinical characteristics, and management of extra‐thyroidal organ‐specific and systemic ADs co‐existing with thyroid cancer.

In this study, we evaluated patients with DTC who underwent iodine‐131 (^131^I) treatment at our center, specifically analyzing the impact of extrathyroidal ADs on the clinical features of thyroid cancer, the efficacy of ^131^I therapy, and thyroid‐stimulating hormone (TSH) inhibition therapy. The findings aim to enhance the clinical management and treatment strategies for DTC patients with ADs.

## 2 CLINICAL DATA

### 2.1 Research participants

In this retrospective study, a total of 1832 patients with DTC and treated at the Department of Nuclear Medicine of Shanxi Bethune Hospital from January 2019 to September 2022 were initially considered. Following screening, 181 patients with DTC were selected for inclusion. The clinical information of all patients was collected, including age of onset, gender, duration from diagnosis of ADs to the occurrence of thyroid cancer, tumor node metastasis (TNM) characteristics of DTC, benign thyroid disease (including HT and nodular goiter), ^131^I therapy efficacy, levothyroxine dosage, and other relevant clinical data. This study received ethical approval from the Ethics Committee of Shanxi Bethune Hospital (Approval No.: YXLL‐2023‐130) and adhered strictly to the principles outlined in the Declaration of Helsinki. Written informed consent was obtained from all participants before their inclusion in the study.

### 2.2 Inclusion criteria

(1) Patients who underwent either complete or subtotal thyroidectomy along with central lymph node dissection and/or selective cervical lymph node dissection.
(2) Diagnosis of DTC confirmed through pathological examination (including PTC and follicular thyroid carcinoma).
(3) Patients with TSH levels greater than 30 uIU/mL before receiving ^131^I therapy, with comprehensive data available, including serological findings and imaging records throughout the follow‐up period.
(4) Patients who were followed up for at least 6 months following their final ^131^I treatment.

### 2.3 Experiment equipment and reagents

Instrument: Roche Cobase automatic electrochemiluminescence immunoassay (ECLIA) analyzer. Reagents: Roche thyroglobulin (Tg), thyroglobulin antibody (TgAb), and TSH kit (Roche Diagnostics GmbH).

### 2.4 Method

#### 2.4.1 Grouping

A total of 181 patients with DTC were split into the combination group (*n* = 35) and the noncombination group (*n* = 146) based on the presence or absence of ADs, respectively.

#### 2.4.2 Serological tests and imaging examination

Serological quantification measurements of Tg, TgAb, and TSH were conducted using ECLIA (Roche, E170). These tests were performed before 131I therapy and at 3, 6, and 12 months posttherapy. Imaging examinations were conducted as clinically indicated, including ultrasound of the thyroid, cervical and supraclavicular lymph nodes, chest computed tomography (CT), whole‐body bone scintigraphy using technetium‐99m methylene diphosphonate, and whole‐body positron emission tomography (PET)/CT using fluorine‐18 fluorodeoxyglucose (^18^F‐FDG).

#### 2.4.3 ^131^I treatment

The patients were instructed to discontinue thyroid hormone medications for 3–4 weeks before treatment to achieve serum TSH levels >30 μIU/mL. The administered dosage of ^131^I ranged from 100 to 200 mCi depending on the presence of local residue, metastasis, and distant metastasis. Whole‐body iodine scans and localized single‐photon emission computed tomography (SPECT)/CT imaging were performed on the third‐day posttreatment. The preparation and protocol development of ^131^I treatment followed the 2015 American Thyroid Association (ATA) guideline^16^ and the 2021 Chinese ^131^I guidelines for the treatment of DTC.^17^

#### 2.4.4 Reaction to treatment

Patient responses to ^131^I therapy were assessed based on the 2015 edition of the ATA guidelines, and were classified into Excellent Response, Indeterminate Response, Biochemical Incomplete Response, and Structural Incomplete Response.^16^

#### 2.4.5 Follow‐up after treatment

The initial follow‐up was conducted 1–3 months after ^131^I therapy, and serological (Tg, TgAb, TSH) measurement and imaging (color ultrasound, chest CT, whole body bone scintigraphy, etc.) were performed at least 6 months following ^131^I therapy. The efficacy of ^131^I therapy was assessed utilizing a combination of 18F‐FDG‐PET/CT imaging. At the same time, the risk of real‐time recurrence was classified based on a dynamic assessment to adjust levothyroxine dosage and subsequent treatment.^16^ For patients who had a second surgery, the postoperative pathology served as the diagnostic basis for structural lesions. Patients were monitored for a minimum of 6 months after their final ^131^I therapy.

### 2.5 Statistical analysis

Statistical analysis was conducted using SPSS 26.0 and R 4.0.3. Qualitative data presented as the number of cases (%), and the between‐group comparison was performed using the chi‐squared test, the Fisher's exact probability method, or the Wilcoxon rank sum test for the comparison of two independent samples. The normality test was performed using the Shapiro–Wilk test, and the homogeneity test was conducted using Levene's test. For quantitative data conforming to normality and homogeneity of variance, the mean ± standard deviation (x̅ ± s) was used for statistical description, and the *t*‐test of two independent samples was used for the comparison between groups. For quantitative data that did not satisfy the requirements, M (P25‐P75) was used for statistical description, and the Wilcoxon rank sum test was employed for comparison between groups. The receiver operating characteristic (ROC) curve was used to calculate the optimal cut‐off value, and the balance of baseline demographic characteristics propensity score matching. The inspection level α was .050.

## 3 RESULTS

### 3.1 Baseline characteristics

This study included a total of 181 patients, among whom 35 had DTC coexisting with AD. During the study period, approximately 2% of patients diagnosed with DTC in our department received ^131^I therapy. Among the cases reviewed, there were eight cases of RA, eight cases of psoriasis, five cases of ankylosing spondylitis, three cases of SS, two cases of SLE, and one case each of giant cell arteritis, ulcerative colitis, autoimmune hepatitis, dermatomyositis, myasthenia gravis, Takayasu arteritis, antisynthetase antibody syndrome, scleroderma, and vitiligo (Table 1).

**Table 1:** The clinical data of 35 DTC patients with ADs.

No.	Gender	Age (years)	ADs	The course of ADs before the diagnosis of DTC	Immunosuppressants or hormonal medications(yes or no)
1	F	57	Rheumatoid arthritis	1 year	Yes
2	F	47	Psoriasis	30 years	Yes
3	M	70	Ankylosing spondylitis	20 years	Yes
4	F	55	Ankylosing spondylitis	6 months	No
5	M	55	Ankylosing spondylitis	5 months	No
6	F	60	Sjogren's disease	4 months	No
7	F	44	Rheumatoid arthritis	7 years	Yes
8	F	52	Systemic lupus erythematosus	13 years	Yes
9	M	57	Psoriasis	7 years	No
10	M	77	Giant cell arteritis	6 months	Yes
11	F	42	Scleroderma	10 months	No
12	M	55	Psoriasis	40 years	No
13	F	59	Psoriasis	1 year	Yes
14	M	56	Rheumatoid arthritis	3 years	Yes
15	F	48	Ulcerative colitis	16 years	Yes
16	F	41	Systemic lupus erythematosus	10 years	Yes
17	F	61	Dermatomyositis	5 months	Yes
18	M	34	Vitiligo	7 years	No
19	F	32	Myasthenia gravis	10 years	Yes
20	F	41	Autoimmune hepatitis	3 years	No
21	F	51	Ankylosing spondylitis	6 years	Yes
22	M	37	Ankylosing spondylitis	10 years	Yes
23	M	58	Rheumatoid arthritis	3 years	Yes
24	M	63	Psoriasis	20 years	Yes
25	F	51	Rheumatoid arthritis	3 years	Yes
26	F	54	Psoriasis	10 years	No
27	F	28	Psoriasis	10 years	No
28	F	56	Rheumatoid arthritis	7 months	No
29	F	30	Takayasu's arteritis	6 months	Yes
30	F	57	Rheumatoid arthritis	4 years	Yes
31	M	44	Antisynthetase antibody syndrome	2 years	Yes
32	F	53	Rheumatoid arthritis	9 years	Yes
33	M	56	Psoriasis	20 years	Yes
34	F	61	Sjogren's disease	3 months	No
35	F	49	Sjogren's disease	2 years	No

Abbreviations: AD, autoimmune disease; DTC, differentiated thyroid cancer.

The male‐to‐female ratio was 1:2. The median age of the patients was 53.5 years, and the time from the diagnosis of AD to the detection of thyroid cancer ranged from 3 months to 40 years, with a median duration exceeding 6.5 years. The comparison of baseline demographic characteristics between groups revealed that age (*t* = −2872, *p* < .01) was substantially different between the combination and noncombination groups. ROC curve analysis was employed to determine the appropriate cut‐off value for age, yielding an area under the curve of 0.654 (95% confidence interval: 0.557–0.751, *p* < .01) and an optimal cut‐off value of 50.5 years. No significant differences in age classification and gender were observed between the combination and noncombination groups (*p *> .05) (Table 2).

**Table 2:** Comparison of baseline demographic characteristics.

Indicators	Non‐ADs group (*N* = 146)	ADs group (*N* = 35)	*t*/χ²$\chi^{2}$	*p* Value
Age (years)	45.01 ± 12.08	51.48 ± 11.29	−2.872	.005
Age classification			0.056	.812
<50.5 years	99 (67.81)	23 (65.71)		
≥50.5 years	47 (32.19)	12 (34.29)		
Gender			0.056	.812
Male	47 (32.19)	12 (34.29)		
Female	99 (67.81)	23 (65.71)		

Abbreviation: AD, autoimmune disease.

### 3.2 Propensity score matching

Propensity score matching was performed using age as the matching factor, with a ratio of 1:3 between the two groups. This process resulted in a total of 112 matched patients, comprising 32 (28.57%) cases with ADs and 80 (71.42%) cases without ADs. There were no statistically significant differences in the baseline demographic characteristics across groups after propensity score matching (*p *> .05) (Table 3).

**Table 3:** Comparison of baseline demographic characteristics following propensity score matching.

Indicators	Non‐ADs group (*N* = 80)	ADs group (*N* = 32)	*t*/$\chi^{2}$	*p* Value
Age (years)	48.30 ± 10.36	50.38 ± 10.79	−0.947	.346
Age classification			1.033	.309
<50.5 years	41 (51.25)	13 (40.63)		
≥50.5 years	39 (48.75)	19 (59.38)		
Gender			0.143	.705
Male	28 (35.00)	10 (31.25)		
Female	52 (65.00)	22 (68.75)		

Abbreviation: AD, autoimmune disease.

### 3.3 Analysis of clinical features after propensity score matching

The comparison of clinical features between groups revealed a statistically significant difference in levothyroxine/weight (ug/kg) between the combination and noncombination groups (*u* = 2.168, *p* < .05). The patients with DTC having ADs received a larger dose of levothyroxine than patients with DTC without ADs (Table 4).

**Table 4:** Comparison of clinical features after propensity score matching.

Indicators	Non‐ADs group (*N* = 80)	ADs group (*N* = 32)	*u*/$\chi^{2}$	*p* Value
Number of lesions (*n*, %)			0.015	.901
Single	29 (36.25)	12 (37.50)		
Multiple	51 (63.75)	20 (62.50)		
Long diameter line (cm)	1.20 (0.90, 1.50)	1.40 (0.80, 2.10)	0.223	.823
Capsular invasion (*n*, %)			0.293	.589
No	43 (53.75)	19 (59.38)		
Yes	37 (46.25)	13 (40.63)		
Extrathyroidal invasion (*n*, %)			0.101	.750
No	67 (83.75)	26 (81.25)		
Yes	13 (16.25)	6 (18.75)		
Cancer embolus (*n*, %)			‐	>.999
No	78 (97.50)	32 (100.00)		
Yes	2 (2.50)	0 (0.00)		
N region (*n*, %)			‐	.551
0	13 (16.25)	3 (9.38)		
1	67 (83.75)	29 (90.63)		
Extramembrane invasion of lymph nodes (*n*, %)			‐	.492
No	79 (98.75)	31 (96.88)		
Yes	1 (1.25)	1 (3.13)		
Number of lymph nodes (Median [Q1, Q3])	5.00 (1.00, 10.00)	3.00 (1.00, 9.00)	−0.488	.625
Total number of lymph nodes (Median [Q1, Q3])	18.50 (7.00, 39.75)	13.00 (6.00, 27.00)	−1.108	.268
Ratio of lymph nodes (Median [Q1, Q3])	27.67 (11.66, 46.25)	33.33 (7.69, 52.94)	0.890	.374
Cancerous node (*n*, %)			‐	.468
No	74 (92.50)	28 (87.50)		
Yes	6 (7.50)	4 (12.50)		
Distant metastasis (*n*, %)			‐	.740
No	72 (90.00)	28 (87.50)		
Yes	8 (10.00)	4 (12.50)		
Combined with Hashimoto's thyroiditis (*n*, %)			2.567	.109
No	66 (82.50)	22 (68.75)		
Yes	14 (17.50)	10 (31.25)		
Combined with nodular goiter (*n*, %)			1.166	.280
No	41 (51.25)	20 (62.50)		
Yes	39 (48.75)	12 (37.50)		
Euthyrox/weight (ug/kg) (Median [Q1,Q3])	1.56 (1.38, 1.76)	1.70 (1.52, 2.05)	2.168	.030

*Note*: Used the Fisher's exact probability method.

Abbreviation: AD, autoimmune disease.

### 3.4 Efficacy assessment after propensity score matching

The comparison of ^131^I therapy efficacy between groups revealed no statistically significant difference between the two groups (*p* > .05) (Table 5).

**Table 5:** Prognosis comparison following propensity score matching.

Indicators	Non‐ADs group (*N* = 80)	ADs group (*N* = 32)	*u*	*p* Value
Outcome			1.491	.136
Structured lesions	13 (16.25)	7 (21.88)		
Biochemical response insufficiency	4 (5.00)	3 (9.38)		
Uncertain response	10 (12.50)	6 (18.75)		
Good response	53 (66.25)	16 (50.00)		

Abbreviation: AD, autoimmune disease.

### 3.5 Subgroup analysis

Subgroup analysis of levothyroxin/weight (ug/kg) revealed a statistically significant difference between the two groups in patients aged ≥50.5 years (*u* = 2.603, *p* < .01) (Table 6).

**Table 6:** Subgroup comparison of Euthyrox/weight (ug/kg) following propensity score matching.

Indicators	Non‐ADs group (*N* = 80)	ADs group (*N* = 32)	*u*	*p* Value
Age ≥50.5 years				
Euthyrox/weight (ug/kg)	1.49 (1.33, 1.65)	1.70 (1.52, 2.05)	2.603	.009
Age <50.5 years				
Euthyrox/weight (ug/kg)	1.64 (1.48, 1.92)	1.70 (1.51, 2.01)	0.557	.578
Gender: male				
Euthyrox/weight (ug/kg)	1.59 (1.50, 1.94)	1.67 (1.58, 2.12)	0.108	.109
Gender: female				
Euthyrox/weight (ug/kg)	1.53 (1.33, 1.74)	1.72 (1.44, 2.05)	1.923	.055

Abbreviation: AD, autoimmune disease.

## 4 DISCUSSION

ADs are conditions characterized by abnormal immune responses directed against the body's own tissues, stemming from dysregulation within the immune system. Chronic inflammation subsequent to autoimmune pathology has sparked considerable interest in assessing the interplay between ADs and cancer. While thyroid‐specific ADs, notably HT, have been frequently implicated in increasing the risk of thyroid cancer, the association between nonthyroid ADs and thyroid malignancy has not been extensively investigated. In this retrospective study, we focused on patients with DTC concurrent with extrathyroidal ADs, aiming to primarily assess the influence of ADs on the pathological characteristics of DTC and the efficacy of ^131^I treatment. Our objective was to provide data to support clinical work.

Based on data from a Chinese study of 278 patients with malignancies and ADs, RA, HT, psoriasis, and immune thrombocytopenia are the four most prevalent ADs.^18^ Both meta‐analyses and observational studies have consistently revealed an increased risk of thyroid cancer in patients with various ADs such as RA,^10^ SLE,^11^ SS,^12^ inflammatory bowel disease,^13^ and vitiligo.^14^ Consequently, it appears conceivable that the presence of ADs may have a notable influence on the development of thyroid cancer. Notably, findings from a nationwide group study conducted in Korea corroborate these observations, demonstrating an increase in the incidence rates and risk of thyroid cancer in patients with ADs. Specifically, the association observed between local ADs such as vitiligo, inflammatory bowel disease, and RA, and the increased risk of thyroid cancer is particularly significant.^15^ In this study, patients more often had complications such as RA and psoriasis, followed by ankylosing spondylitis, SS, and SLE, which is consistent with previous studies. Patients were typically administered immunosuppressants or hormones throughout the course of their disease. While such treatments have the potential to decrease the immune function, they also pose a risk of predisposing patients to secondary infections or possibly fostering tumorigenic processes.^19^ However, in a recent group study involving 478,753 participants from the UK Biobank, a contrasting observation was discovered. Despite establishing a significant association between immune‐mediated diseases and increased risk of cancer, the study found no discernible link between AD and the risk of thyroid cancer.^20^

The patients with DTC and concurrent ADs in this study tended to be diagnosed at an older age compared to those with DTC alone. This trend could potentially be attributed to the increasing emphasis on thyroid‐related disorders and the incorporation of thyroid ultrasound into routine medical assessments. Although the male‐to‐female ratio among patients having DTC with AD (1:2) exceeded that of patients with thyroid cancer alone (1:3), no statistically significant difference in gender distribution was observed between the two groups.^1^ Given the small sample size and the presence of multiple concurrent ADs, the analysis of clinical characteristics within each group was significantly influenced by confounding factors. Consequently, to reduce potential biases stemming from baseline dissimilarities, age stratification was used, followed by propensity score matching analysis. The outcomes revealed that age‐related disparities were rendered inconsequential between patients with or without ADs. Hence, we concluded that there was no difference in baseline characteristics between patients having DTC with ADs and those without ADs. The patients in this study had ADs many years before tumor diagnosis, which is consistent with the findings of Fan et al., who considered that the type of ADs is related to the sequence of tumor diagnosis, and that RA and psoriasis typically developed before the diagnosis of tumors.^18^ Another study reported that only 16% of malignancies preceded rheumatic immune system diseases, while 40% and 44% occurred concurrently with or after rheumatic immune system diseases, respectively.^21^

To further assess the impact of ADs on the clinical characteristics of patients with DTC, we identified the optimal cut‐off value for age using ROC curve analysis. This cut‐off value facilitated age‐based subgrouping for the propensity score matching analysis. The findings indicated that ADs did not affect the number of primary lesions, local invasion, lymph node stage, distant metastasis, or other clinical features in patients with DTC. Therefore, ADs do not promote the progression of DTC. Conversely, numerous animal experiments and clinical studies examining the relationship between autoimmune thyroiditis and thyroid cancer indicate that ADs within thyroid tissue may exert a protective effect on the occurrence and development of thyroid cancer. However, there is a lack of literature addressing the impact of extrathyroidal ADs on the development and the occurrence of thyroid cancer. A mouse model‐based experimental investigation that examined the association between autoimmune thyroiditis and PTC revealed that, while concurrent thyroiditis with PTC had minimal impact on its progression, pre‐existing thyroiditis significantly reduced the incidence and severity of PTC.^22^ Meta‐analysis of clinical data also indicates that HT raises the risk of PTC and slows its progression.^23^ Patients with DTC exhibiting HT have a smaller tumor diameter, which is possible due to the cytokines released by infiltrating lymphocytes suppressing or limiting tumor growth. There is no difference in age, multifocality, nodal stage, membrane, and extramembrane invasion, or distant metastasis.^5^, ^20^, ^24^

The results from this study also demonstrated that DTC was diagnosed in patients with ADs at an older age; however, this did not impact the TNM features of the tumors. Therefore, we hypothesize that thyroidal and extrathyroidal ADs have protective effects on the incidence of thyroid cancer—they may postpone the incidence of thyroid cancer but have no influence on its progression.

In our analysis of clinical characteristics, we observed that patients with DTC and comorbid ADs required a higher unit dose (μg/kg) of levothyroxine compared to those without comorbid ADs. This difference was particularly notable among elderly female patients with ADs, who required a higher unit dose of levothyroxine. Given the absence of differences in TNM staging or response to ¹³¹I therapy between the two groups, we cannot attribute the higher levothyroxine dosage in patients with DTC having ADs to disease severity or suboptimal ¹³¹I therapy response. Previous research has established that autoimmune thyroiditis does not impact the efficacy of ¹³¹I therapy in patients with DTC, which are in line with our findings.^25^ Despite the relatively small sample size of this study, the preliminary results provide valuable clinical data. Based on our findings, extrathyroidal ADs appear to have no significant impact on ¹³¹I therapy outcomes in patients with DTC, hence, special attention is not required.

This study has several limitations. First, data on whether DTC affects the stability of ADs were not collected or indicated, which presents an area for further research. Second, as a retrospective study, the limited sample size may compromise the accuracy of the statistical analysis and obscure the impact of ADs on the clinical characteristics and therapeutic efficacy in patients with DTC.

## 5 CONCLUSION

Extrathyroidal ADs may facilitate the diagnosis of DTC in elderly women; however, no significant effects on the clinical characteristics of thyroid cancer or on the efficacy of ¹³¹I therapy were observed. Although ADs may increase the unit dose of levothyroxine required by patients with DTC, this increase is not associated with the severity or condition of the patient. Consequently, nuclear medicine physicians need not place special emphasis on the presence of ADs when developing treatment plans for patients with DTC.

## AUTHOR CONTRIBUTIONS

**Ya‐hong Long**: Formal analysis; resources; writing—original draft; writing—review and editing. **Na Li**: Data curation; formal analysis; resources; writing—review and editing. **Le Ma**: Conceptualization; data curation; formal analysis; resources. **Wan‐chun Zhang**: Conceptualization; data curation; resources; writing—original draft; writing—review and editing.

## CONFLICT OF INTEREST STATEMENT

The authors declare no conflict of interest.

## ETHICS STATEMENT

This study was conducted with approval from the Ethics Committee of Shanxi Bethune Hospital (NO: YXLL‐2023‐130). This study was conducted in accordance with the declaration of Helsinki. Written informed consent was obtained from all participants.

## Data Availability

The data sets used and/or analyzed during the current study available from the corresponding author on reasonable request.
